# The Multivariable Multiaxial Suggestibility Inventory-2 (MMSI-2): A Psychometric Alternative to Measure and Explain Supernatural Experiences

**DOI:** 10.3389/fpsyg.2021.692194

**Published:** 2021-07-16

**Authors:** Álex Escolà-Gascón, Neil Dagnall, Josep Gallifa

**Affiliations:** ^1^School of Psychology, Education and Sport Sciences, Blanquerna, Ramon Llull University, Barcelona, Spain; ^2^Faculty of Health, Psychology and Social Care, Manchester Metropolitan University, Manchester, United Kingdom

**Keywords:** delusions, anomalous perceptions, anomalous phenomena, structural equation modeling, paranormal beliefs

## Abstract

This paper presents the English adaptation of the *Multivariable Multiaxial Suggestibility Inventory-2* (MMSI-2), a questionnaire developed specifically for psychological assessment and prediction of anomalous phenomena. The sample consisted of 613 respondents from England (47.6% were women and 52.4% men). All of them were of legal age (mean = 34.5; standard deviation = 8.15). An *exploratory factor analysis* was applied, and three *confirmatory factor models* were adjusted. *Omega* coefficients and *test-retest* designs were used for reliability analysis. The MMSI-2 has a valid internal structure consisting of five macrofactors: *Clinical Personality Tendencies* (CPT), *Anomalous Perceived Phenomena* (APP), *Incoherent Manipulations* (IMA), *Altered States of Consciousness* (ASC), and *Openness* (OP). Omega coefficients for CPT and OP factors were low but acceptable. Furthermore, *test-retest* trials were excellent for all scales and factors. The psychological factors CPT, IMA, and ASC predicted 18.3% of the variance of anomalous experiences (APP). The authors concluded the English MMSI-2 was a valid and reliable test for the evaluation of anomalous phenomena but recommend that subsequent research reviews the predictive quality of the underlying model.

## Introduction

Anomalous phenomena represent behaviors and perceptions that conflict with the ontological bases of current science (e.g., Gallagher et al., 1994; Kuhn et al., 2016). Examples are paranormal beliefs and experiences, such as feeling the physical presence of deceased beings, and hearing unexplained noises or blows (e.g., Jinks, 2019). Other parapsychology perceptions include anticipation of unpredictable stimuli (called precognition), mind-and-mind communication (telepathy), and mind-matter interaction (e.g., Wiseman and Watt, 2017; Cardeña, 2018). These experiences constitute rationally impossible phenomena in scientific terms (e.g., Tobacyk, 2004; Musella, 2005). Accordingly, psychology and psychiatry generally explain these behaviors and phenomena from three theoretical approaches, both clinical and subclinical (e.g., Escolà-Gascón, 2021).

The first relates to the *continuum* model *of psychosis* (e.g., Johns and van Os, 2001; van Os et al., 2009; American Psychiatric Association, 2013). From this perspective, anomalous phenomena are explained as hallucinatory symptoms, which manifest at different levels (e.g., Stefanis et al., 2002; Shapiro et al., 2019). Less intense or attenuated hallucinations represent subclinical symptoms that lack psychopathological value within the framework of psychosis (e.g., Nordgaard et al., 2019; Fekih-Romdhane et al., 2020). The most frequent and invasive hallucinations are the most dysfunctional and define acute hallucinatory pictures (in clinical terms) (e.g., Kelly et al., 2020). The fact that anomalous phenomena are classified as hallucinations means that they are not real and lack ontological value (e.g., Reber and Alcock, 2020).

The second approach relates to *perceptual distortion* and *deception* (e.g., Ey et al., 1980). Although both perceptual alterations present within the spectrum of psychoses, they differ from hallucinations since they require a sensory triggering object (e.g., Jaspers, 1993; El-Mallakh and Walker, 2010). However, perceptual deception does not usually have a psychopathological origin (e.g., Parker, 2006), which is why they are also called “illusions” or perceptual biases (e.g., Barberia et al., 2013, 2018). Examples include *pareidolia* and the *Barnum effect* and affective (e.g., Belloch et al., 1995; Shermer, 2011). Numerous studies have found that these distortions of perception are common in subjects who believe in the existence of the paranormal (e.g., Matute et al., 2011; Griffiths et al., 2018; Torres et al., 2020). Likewise, in some cases, they represent causal attributions or illusions that try to reduce levels of uncertainty in the face of specific problems, so that their psychological function responds to the need to seek control (e.g., Groth-Marnat and Pegden, 1998; Matute et al., 2015).

The third model is called *phenomenological* and *cognitive* because it focuses on the belief systems and meanings of the individual (e.g., Irwin, 1993, 2009, 2017; Font, 2016; Irwin and Marks, 2018; Lange et al., 2019). According to this model, human beings interact with environmental *inputs* through neuropsychological processes that define the sensation and perception of stimuli (e.g., Wain and Spinella, 2007). These conclude with the cognitive representation of perceived objects (e.g., Lee et al., 2018). The mental representation of a given content implies the conscious attribution of a category or meaning - previously learned and recorded - which allows the individual to develop a logical and relational interpretation of the phenomena that occur in objective reality (e.g., Fishbein and Ajzen, 1975).

Interpretations configure the belief system and allow for a conscious sense of experience (e.g., Irwin et al., 2013). Note that from this perspective, the concept of “belief” does not mean accepting the existence or non-existence of an object; it refers to the way of understanding environment inputs (e.g., Drinkwater et al., 2017). Representations are variable, and each individual constructs their own comprehensive schemes on the functioning of reality (e.g., Pennycook et al., 2012). The crystallization of learned schemes form the belief systems (e.g., Schriever, 2000; Irwin, 2009). Then, the “paranormal” experience is resolved psychologically by explaining it as a cognitive representation, whose categories or meanings are based on contents incompatible with scientific rationalism (e.g., Simmonds-Moore, 2016).

However, psychology has the problem that certain scientific investigations have tried to statistically contrast the occurrence of some apparently impossible experiences and obtained significant results. This is the case for pre-cognition (e.g., Tressoldi et al., 2009; Bem, 2011; Mossbridge et al., 2012; McCraty and Atkinson, 2014; Bem et al., 2016; Mossbridge and Radin, 2018), telepathy (e.g., Moss and Gengerelli, 1967; Krippner and Ullman, 1970; Honorton, 1985; Sheldrake and Avraamides, 2009), the anomalous reception of information or mediumship (e.g., Beischel and Schwartz, 2007; Kelly and Arcangel, 2011; Sudduth, 2013; Beischel et al., 2015), and the mind-matter interaction (e.g., Radin, 2006; Tressoldi et al., 2014). Studies of core “psi” phenomena experiences (see Cardeña, 2018; Jinks, 2019), such as these facilitate discussion regarding the possibility of the existence of alternative phenomena that transgress the bases of human perception (e.g., Utts, 2018; Cardeña, 2019). For this reason, these experiences are also called *anomalous*, since results are observed in favor of these phenomena that supposedly challenge the foundations of science (see French and Stone, 2014). This is a problem because if, until now, paranormal experiences were discussed and examined as hallucinations, perceptual deformations and representation of meanings.

These studies were highly controversial, and currently, the scientific value of the respective results is contentious (e.g., Reber and Alcock, 2020). It seems that the scientific community is divided into two factions (see Carter, 2012). Model 1 starts from the apriorism that “psi” phenomena exist and represents phenomena with ontological-scientific validity (see Bem, 2011). Whereas, model 2 contends that such phenomena do not exist (see also Álvarez, 2007). This last position produces research that systematically and rationally denies the existence of “psi” (e.g., Carter, 2012). Since both lines have published research - and even meta-analysis (e.g., Storm et al., 2010, 2013; Utts, 2018) - supporting their perspectives, no unanimous conclusion has been reached (e.g., French and Stone, 2014). It is common for model 1 scientists to not recognize the research of model 2 scientists, and vice versa (e.g., Carter, 2012). Moreover, this controversy is so competitive that some scientists overlook formal scientific research, discussions and databases published on this topic (see Moreira-Almeida et al., 2005; Parker, 2006). This is a serious error since science can be harmed by erroneous research decisions. Explicitly, researchers cannot and should not ignore the controversy associated with the complexity of knowledge (e.g., Bunge, 2013). The complexity of *knowledge* must be resolved empirically and rationally through the application of the scientific method, and not via arguments based on opinion that aise from academic beliefs-conceptions (e.g., León and Montero, 2002).

This discussion on how to interpret “psi” or anomalous phenomena directly impacts psychological evaluation (within and outside the psychopathological field) (e.g., Escolà-Gascón and Gallifa, 2020), since in numerous clinical cases behaviors similar to “psi” phenomena are described (e.g., Bobrow, 2003). How is a hallucination different from a “parapsychological” experience (related to “psi” phenomena)? The answer to this question is resolved to the extent that mental health professionals believe in the existence of “psi” phenomena. Professional disinformation is also a fact, and subsequently individuals rely heavily on their own opinion or belief (e.g., Pasricha, 2011). Outside the experimental context, there are no psychiatric and psychological assessment tools that address this conflict. Specifically, psychometric questionnaires can be found that measure anomalous phenomena such as hallucinations (e.g., Stefanis et al., 2002; Mason and Claridge, 2006; Fonseca-Pedrero et al., 2011), perceptual deformations (e.g., Chapman et al., 1978; Bell et al., 2006) or illusions (e.g., Peters et al., 2004), or evaluate these behaviors as if phenomena exists (e.g., Wahbeh et al., 2019). Actually, neither the apriorism of model 1, nor the apriorism of model 2 are determinable, because in such an instance the *Aristotelian fallacy of affirming the consequent* applied to statistical methodology is incurred (see Pardo and Román, 2013): one would be accepting the veracity of a hypothesis (null or alternative) from causes that have been contrasted, but remain uncertain because results are contradictory.

What consequences would the diagnosis of a hallucination as a “psi” phenomenon and vice versa have for the patient? This question confronts the scientific beliefs of each professional - there are those who offer a discourse close to model 1 and those who defend model 2 -, but in any case, it reflects also a need: that of an *a priori* model that does not deny or affirm the existence of “psi” phenomena. It would be useful to propose an integrative model (not an eclectic one), which allows examination of anomalous phenomena from a *utilitarian*, pragmatic and empirical-statistical *perspective*.

This perspective could be based on the following idea: it is not the job of the psychiatrist or psychologist to contrast the empirical-experimental value of what the patient tells (e.g., Groth-Marnat, 2009). However, it is important to examine whether the anomalous phenomena perceived by the patient could be explained by other psychological indicators usually observed in these cases (e.g., Irwin, 2009; Pasricha, 2011). Discarding the greatest possible number of explanations (or variables) observed from psychology and what is scientifically observed in anomalous phenomena does not resolve the controversy between model 1 and model 2, though, it allows greater objectivity.

This study examined the validity of the internal structure of the empirical-statistical model of the *Multivariable Multiaxial Suggestibility Inventory-2* (*hereafter* MMSI-2). This is a broad-spectrum questionnaire specialized for the evaluation of the psychological foundations of anomalous phenomena, which gathers up to 16 psychological variables predictive of this class (e.g., Escolà-Gascón, 2020a).

The MMSI-2 starts from 4 logical-rational assumptions: (1) a perceptual alteration in itself is not a hallucination, a perceptual deformation, a cognitive bias or a fraudulent invention. (2) The measurement and quantification of other psychological variables (such as structured sources of information) is required to contrast the hallucinatory, perceptive, cognitive, and fraudulent value of a perceptual alteration. (3) Although certain psychological variables do not statistically explain certain anomalous phenomena, it does not mean that these phenomena have a “parapsychological” or “supernatural” origin. Finally, (4) anomalous phenomena can be explained or statistically unexplained, but that does not imply that they are “inexplicable” or “explainable” phenomena for science” (the latter belongs to the field of *philosophy of science* and not to the scientific method) (see Escolà-Gascón, 2020b).

In clinical practice focused on psychiatric evaluation of cases, the experimental method is not applied, and *self-report techniques* mostly inform diagnostic decisions (see Groth-Marnat, 2009). Therefore, what should be offered is not only basic research focused on how to “export” the experimental method to clinical practice, but also on “importing” or developing the necessary evaluation systems that allow objective and effective evaluative decisions (this means, based on evidence).

The objective of this study was to offer a useful tool that allows knowing - from the empirical-statistical evidence - whether there are objective reasons to suspect the presence of psychological indicators that could explain the anomalous experiences reported by the patient, without assuming *a priori* the existence or non-existence of this class of “supposed” phenomena.

## Materials and Methods

### Description of the Sample

The sample consisted of 613 participants (47.6% were women and 52.4% men). All were adults (mean = 34.5; standard deviation = 8.15), who agreed to participate voluntarily in the research, and declared no official psychiatric history. The subjects came from three English locations: 33.8% resided in Portsmouth, 32.1% resided in Worthing, and 34.1% resided in Brighton. In relation to educational level, 32.8% completed secondary education, 36.4% also attended vocational training, and 30.8% reached university studies. Likewise, the participants signed an informed consent form in which the objectives of the study were specified and guaranteed that the treatment of the data would be completely anonymous. Those who accepted (77.6%) offered their first name and email as the only references to contact them and thus were able to develop the *test-retest* design (see section Statistical Analysis Applied). Finally, two conditions were considered as inclusion and exclusion criteria for the sample: (1) all subjects had to be adults over 18 years of age, and (2) no subjects had to have official psychiatric antecedents. All participants met these two conditions.

### Procedure

This research used a correlation-based design grounded in analysis of self-report questionnaires. Specifically, during the summer months between 2016 and 2019, the research team traveled to England for educational and work reasons unrelated to this research. During different stays, the *Multivariable Multiaxial Suggestibility Inventory-2* (MMSI-2) questionnaire was applied digitally, which was originally developed in Spain by Escolà-Gascón (2020a). The translation was carried out by the same author of the questionnaire and was subsequently reviewed by different English-speaking health professionals, both Americans (specifically from the state of California, USA), and British (specifically residents between Brighton and Worthing) (for more information, see the Acknowledgments section).

Originally, the translation was done as a complement to the Spanish version (in the hypothetical case that other foreign professionals wanted to use the MMSI-2). However, the possibility of traveling to England and the United States led to the mobilization of the necessary resources to prepare its application in the respective countries. It was then that professionals who reviewed the translations and collaborated with the research were contacted. The English application of the MMSI-2 during the months and years specified above was carried out in parallel with the Spanish application, which is also in the process of publication.

It should be taken into account that the items of the MMSI-2 were successfully subjected to peer-to-peer validation (in their original version), this allowed for the elimination of 49 items out of the 223 belonging to the first version of the MMSI. In the same way, before proceeding with the applications of the 174 definitive items, an unpublished pilot study was developed that warned of the errors that had to be changed to optimize the initial factorial solutions. These errors were based on excessively ambiguous expressions that prevented obtaining the minimum variability necessary for the application of any statistical analysis. This type of error was corrected. Thus, the 174 final items of the MMSI were distributed in such a way that it was possible to detect if the subject answered randomly to the questions posed. A scale was developed (called *Inconsistencies* or K) with 12 statements that expressed rationally impossible contents (e.g., “Little Red Riding Hood is a real character”). These items were positioned based on question 52, since they intended to prevent not only random responses but also the fatigue effect associated with this type of extensive test (see Barbero et al., 2015).

This study used *confirmatory factor analysis* (hereafter CFA) to validate the empirical model of the 16 primary dimensions of MMSI-2, which were obtained by exploratory factorial techniques. It is precisely for this reason that the underlying structural model of the MMSI can be called “empirical-statistical,” given that – unlike most of the questionnaires that are identified in this context of anomalous phenomena – the scales were not defined from a hypothetical-deductive theory. The published scientific evidence was considered - not scientific “*a priorism*” discussed in the theoretical framework - and the exploratory factor analyses applied to the items of the Spanish version (see Escolà-Gascón, 2020a).

Given that it was intended to test the construct validity of the 16 dimensions of the MMSI, only the direct scores for each scale of the questionnaire were recorded in the raw data matrix. The individual responses for each item were not saved because the application was digital and the correction of the responses was automated to save time in the manual coding of the scores and to increase the sample size. It should be noted that no subject showed missing values, so the sample used did not undergo purifications that substantially reduced its size. The process related to the conceptual and methodological development of the MMSI-2 can be consulted in more detail at Escolà-Gascón (2020a,b).

### Description of the Instrument

The English version of the *Multivariable Multiaxial Suggestibility Inventory-2* (MMSI-2) was used, which consists of 174 items, whose responses are coded using a Likert scale that fluctuates from the value 1 (“completely disagree”) to 5 (“totally agree”). Its items are distributed in the following 16 first-order scales: *Inconsistencies* (K), *Lies* (L), *Fraud* (F), *Simulation* (Si), *Neurasthenia* (Nt), *Substance Use* (Cs), *Suggestibility* (Su), *Thrill-Seeking* (Be), *Histrionism* (Hi), *Schizotypy* (Ez), *Paranoia* (Pa), *Narcissism* (Na), *Anomalous Visual/Auditory Phenomena* (Pva), *Anomalous Tactile Phenomena* (Pt), *Anomalous Olfactory Phenomena* (Po), and *Anomalous Cenesthetic Phenomena* (Pc). The exploratory factor analyses of the Spanish version indicated that these 16 scales could be grouped into 4 higher order factors: *Clinical Personality Tendencies* (CPT), *Anomalous Perceived Phenomena* (APP), *Incoherent Manipulations* (IMA), and *Altered States of Consciousness* (ASC). The MMSI-2 presents statistical evidence in its Spanish version that supports the validity and reliability of the test, even in its reduced version (the MMSI-2-R) (see Escolà-Gascón, 2020a,b; Escolà-Gascón and Gallifa, 2020).

### Statistical Analysis Applied

Data analysis used the JAMOVI program (see The Jamovi Project, 2019) and the *R code* applied to the *R Core team* (see R Core Team, 2018).

Three confirmatory factor analyses (CFAs) were applied by the *maximum likelihood* estimation *method* and were based on: (1) the original Spanish version, (2) second-order factors extracted from a previous *exploratory factorial analysis* (hereafter EFA), and (3) the predictive value of second-order factors on the anomalous phenomena themselves (in this way, the underlying empirical-statistical model could be tested). In the EFA, the criterion based on the *minimum unweighted residuals* was used as the extraction method, since it does not require the *a priori* calculation of the *communalities* of items (see Mulaik, 2018). The *parallel analysis* technique was used to determine the number of factors to be extracted (e.g., Reise et al., 2000) because it is a more precise and effective method than the traditional *Kaiser* criterion (see Kline, 1999). *Direct oblimin* rotation was also applied to optimize the extracted solution. *Orthogonal* rotations were not applied as they are unrealistic criteria in the field of social sciences, since they reduce the correlation between the factors to “0” (see Abad et al., 2015). Logically, the rotation was only applied on the previous EFA, and not on the CFAs.

Reliability was examined for each *macrofactor* by the *internal consistency* coefficients based on factorial loads. They differ from the classic *Cronbach's alpha* in that they do not take into account the number of items of each factor; instead, the factorial loads obtained for each grouped variable are used (e.g., Barbero et al., 2015). For this reason, they are very useful coefficients in the multidimensional measurement of internal consistency (see Trizano-Hermosilla and Alvarado, 2016). There are different coefficients based on factorial loads (see Heise and Bohrnstedt, 1970), but in this study, the version proposed by McDonald (1999) can be formulated as follows:

(1) $$\begin{array}{c} \omega_{t}=\frac{{\left(\sum^{\text{​}}{\text{λ}}_{j}\right)}^{2}}{\left[{\left(\sum^{\text{​}}{\text{λ}}_{j}\right)}^{2}+\sum^{\text{​}}\left(1-{\text{λ}}_{j}^{2}\right)\right]}=\frac{{\left(\sum^{\text{​}}{\text{λ}}_{j}\right)}^{2}}{\left[{\left(\sum^{\text{​}}{\text{λ}}_{j}\right)}^{2}+\left(\sum^{\text{​}}\psi \right)\right]} \end{array}$$

where

λ_*j*_ is the saturation of the item-variable *j*,

$\lambda_{j}^{2}$ is the commonality of the item-variable *j*, and

*ψ* is the unique variance.

This equation is integrated into the JAMOVI program. It should be noted that reliability is applied to second-order factors, which means that the subscripts *j* will not be the items but the scales themselves.

Given that the coefficient *ω*_*t*_ would only be applied to the macrofactors and not to the primary scales, we proceeded with the application of *test-retest trials* in the primary scales (which measure the longitudinal consistency of the scores). These tests could only be applied to 23.2% of the sample (*N* = 142) and 160 total days elapsed between both applications (number of minimum days elapsed since the first application = 150; number of flexible days = 10). The number of flexible days refers to the time each participant had to respond to the second application of the questionnaire. After these 10 days, the participant could no longer answer the second application. Of the 77.6% of the subjects, 54.4% did not answer the second application and left the study or responded outside the deadline. A total of 22.4% of the participants did not want to give their email to follow up and answer the second application. The analysis is performed with Student's *t* distribution and Pearson's correlation linear coefficients. Understanding that it is intended to maintain the null hypothesis in these contrasts and the alternative in the case of correlations, if any *Student's t-test* yielded significant differences, non-parametric tests would also be applied (*Mann-Whitney U*-test). In all analyses, a risk of error of 1% was applied.

## Results

### Exploratory Factor Analysis

**Figure 1:**
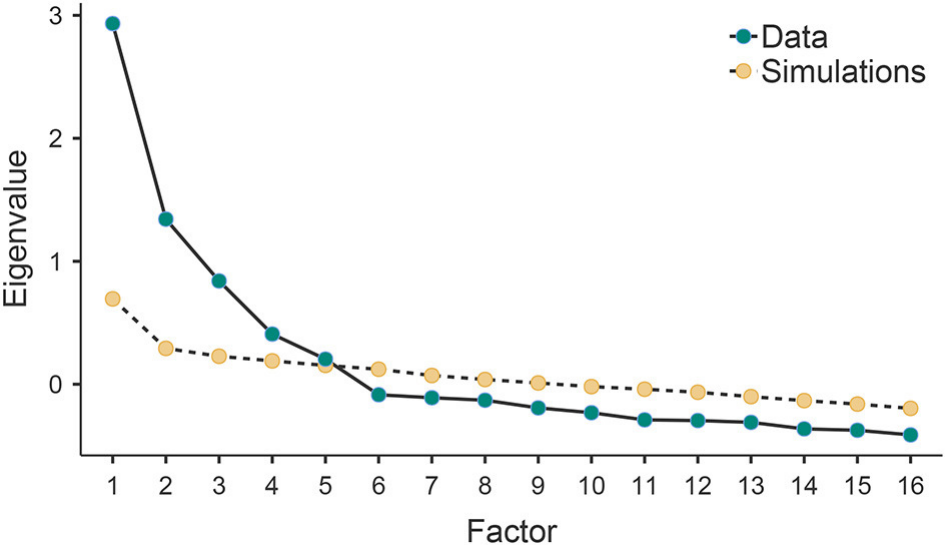
Scree plot of parallel analysis.

Before testing the different models of the internal structure of the MMSI-2, we wanted to explore whether it was possible to extract a factorial solution outside the theoretical background from the raw scores recorded. Table 1 shows the descriptive statistics for each scale. Table 2 shows the exploratory factorial solution obtained, which is formed by five factors that together explain 46.7% of the variance. Considering Figure 1, the crossing of the curves indicates that the best and most stable solution is that which retains up to five factors.

**Table 1:** Descriptive statistics of MMSI-2 scales (*N* = 613).

**MMSI-2 scales**	**Mean**	**Standard deviation**	**Variance**
(K) - Inconsistencies	27.99	9.63	92.65
(L) - Lies	68.31	24.83	616.37
(F) - Fraud	59.37	21.33	454.87
(Si) - Simulation	17.25	6.41	41.11
(Nt) - Neurasthenia	44.83	16.2	262.36
(Cs) - Substance Use	10.64	2.32	5.40
(Su) - Suggestibility	19.29	6.92	47.89
(Be) - Thrill-Seeking	12.03	4.44	19.69
(Hi) - Histrionism	32.29	11.81	139.48
(Ez) - Schizotypy	31.03	10.58	112.00
(Pa) - Paranoia	30.19	11.31	127.91
(Na) - Narcissism	33.46	11.62	135.12
(Pva) - Anomalous Visual/Auditory Phenomena	25.61	9.02	81.44
(Pt) - Anomalous Tactile Phenomena	18.29	6.621	43.84
(Po) - Anomalous Olfactory Phenomena	17.23	6.07	36.84
(Pc) - Anomalous Cenesthetic Phenomena	17.45	5.16	26.60

*MMSI-2, Multivariable Multiaxial Suggestibility Inventory-2*.

**Table 2:** Exploratory factor analysis with oblimin rotation.

**MMSI-2 scales**	**Extracted factors**^**a**^					**Uniqueness**
	**IMA**	**CPT**	**APP**	**OP**	**ASC**	
(K) - Inconsistencies	0.800					0.350
(L) - Lies	0.796					0.375
(F) - Fraud	0.784					0.371
(Si) - Simulation	0.756					0.420
(Ez) - Schizotypy		0.668				0.571
(Hi) - Histrionism		0.612				0.587
(Pa) - Paranoia		0.607				0.635
(Na) - Narcissism		0.553				0.672
(Pt) - Anomalous TactilePhenomena			0.654			0.560
(Pva) - Anomalous Visual/AuditoryPhenomena			0.647			0.604
(Po) - Anomalous OlfactoryPhenomena			0.605			0.611
(Pc) - Anomalous CenestheticPhenomena			0.490			0.751
(Su) – Suggestibility				~1		0.006
(Be) - Thrill-Seeking				0.377		0.751
(Cs) - Substance Use					0.622	0.611
(Nt) - Neurasthenia					0.584	0.642
Explained variance^b^ (%)	15.63	9.69	9.36	7.41	4.65	46.7

*MMSI-2, Multivariable Multiaxial Suggestibility Inventory-2; CPT, Clinical Personality Tendencies; APP, Anomalous Perceived Phenomena; IMA, Incoherent Manipulations ASC, Altered States of Consciousness; OP, Openness*.

a *Loadings under 0.3 were deleted*.

b *Explained variance was taken from the original solution without rotation*.

The first coincides with the factor of the original version called *Incoherent Manipulations* (IMA) and is composed of the K, L, F, and Si scales. The second also coincides with previous research, is called *Clinical Personality Tendencies* (CPT) and is characterized by the Hi, Ez, Pa, and Na scales. The third group includes the Pva, Pt, Po, and Pc scales, which is equivalent to the second-order factor called *Anomalous Perceived Phenomena* (APP). The fourth factor groups the Be and Su scales, a fact that differs from the original statistical justification and proposes the formation of a new second-order factor, which can be called *Openness* (henceforth OP). Both susceptibility (Su) and the search for emotions (Be) represent two facets of personality that describe the subject's predisposition to feel new experiences and tolerate new emotional states (e.g., Costa and McCrae, 2008). This seems to coincide with the “Big Five” model of personality, researched and replicated by multiple studies (see Goldberg, 1993). This will be analyzed in the discussion. The last factor is called *Altered States of Consciousness* (ASC), which includes the Nt and Cs scales. This coincides with previous validations of the MMSI-2.

### Confirmatory Factor Analysis

**Figure 2:**
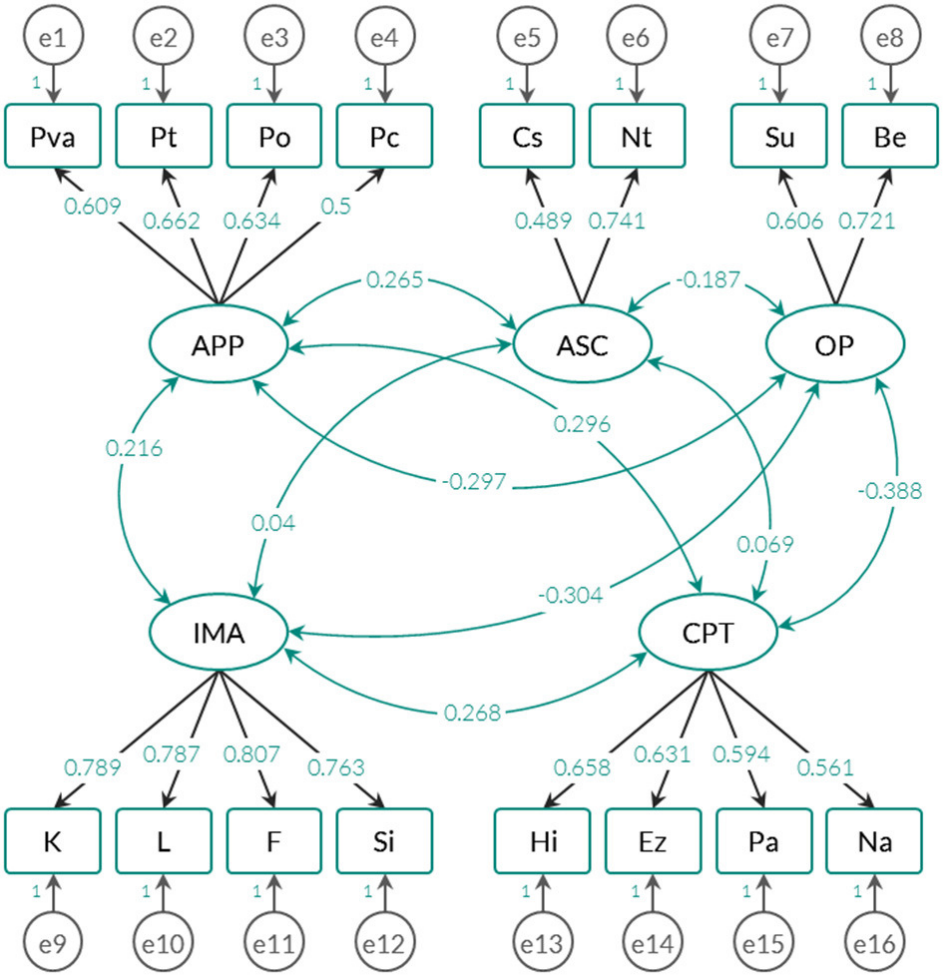
Trace graph for the exploratory-empirical model (5-factor model).

**Figure 3:**
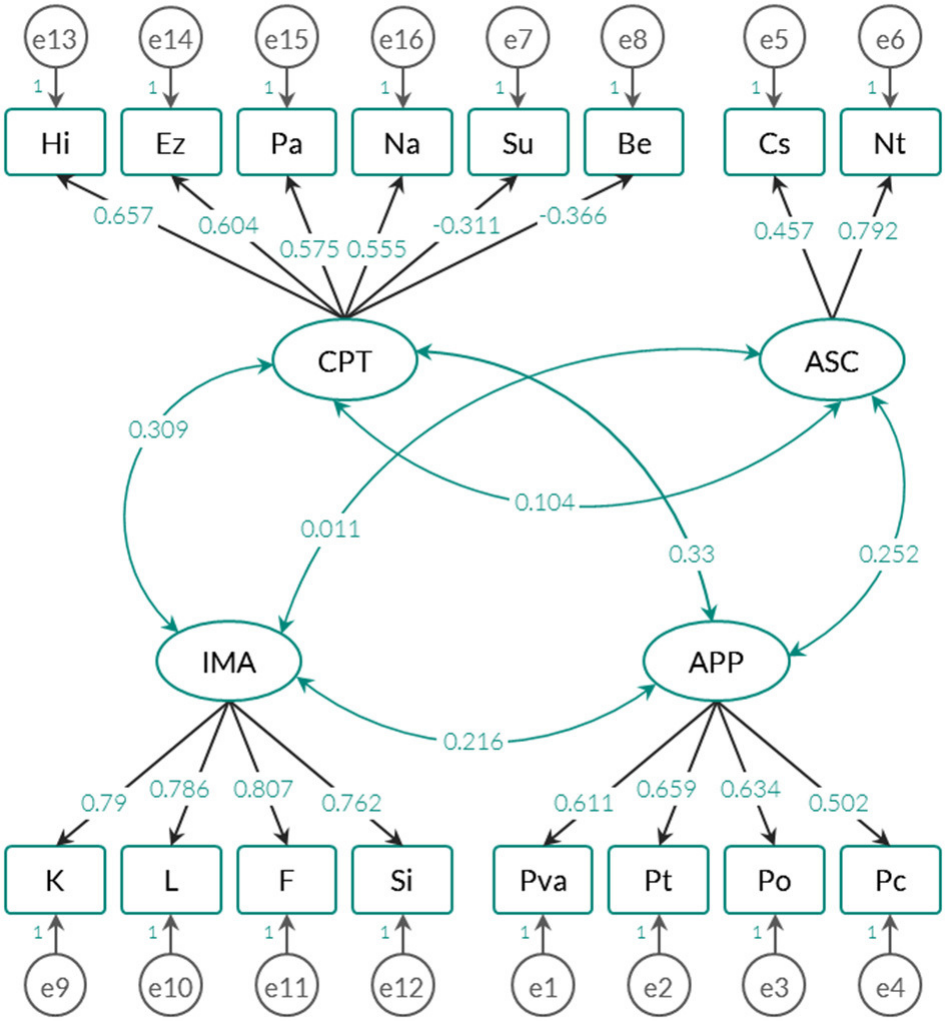
Trace graph of the Spanish theoretical model (4-factor model).

**Figure 4:**
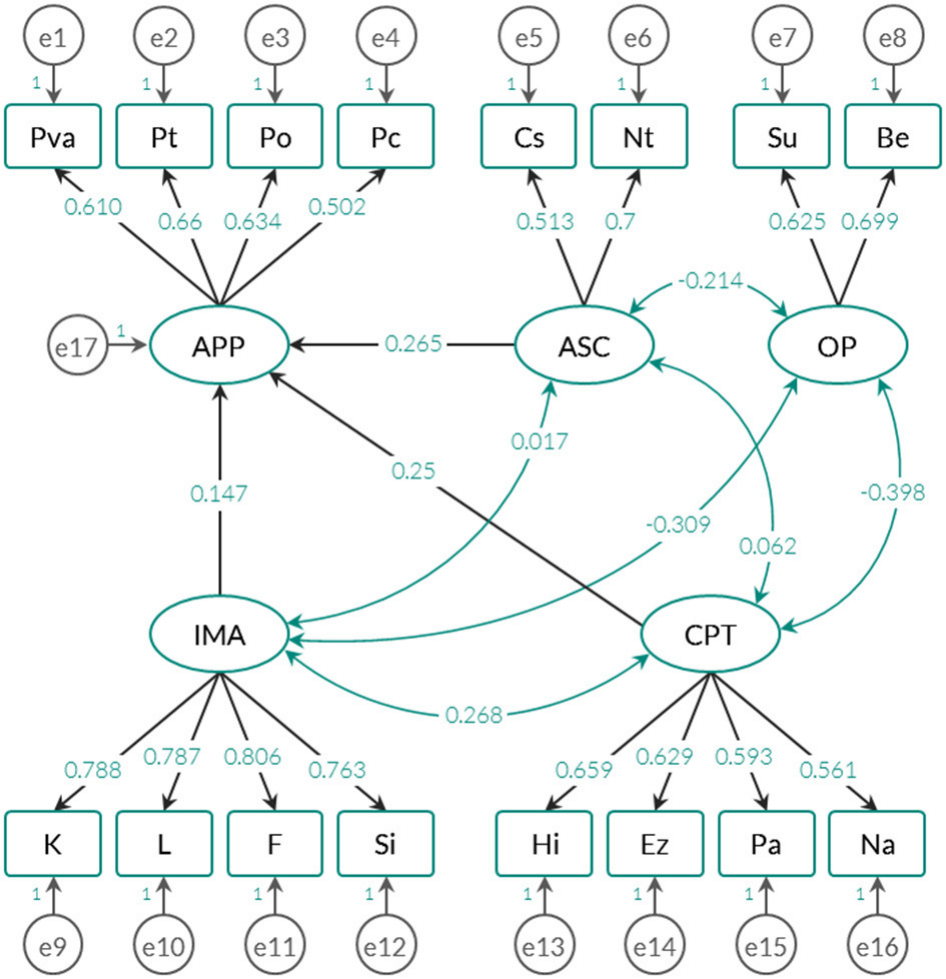
Trace graph of the alternative model (of 5 latent-variables model with 3 factors with prediction effects on APP).

From the previous results, three confirmatory models were adjusted: (1) the *exploratory-empirical model*, which is based on the previous EFA; (2) the *original theoretical model*, whose solution does not include the OP factor (in total, it groups the four factors described in section Description of the Instrument); and (3), the *alternative model* inferred from the first and second models. This last proposal examined with what weights the anomalous phenomena evaluated in the APP factor can be predicted by the IMA, CPT, and ASC factors (this idea is also proposed in the original statistical justification). The weights and standardized correlations for each type of model are shown in Figures 2–4.

To contrast whether the estimated parameters successfully reproduce the variance-covariance matrices extracted from the raw matrix, the fit indices specified in Table 3 were used. It should be noted that the risk of error is adjusted to 1% and that the *Chi Square* statistic is highly sensitive to the sample size (see Brown, 2015). Table 3 also shows the results of the fit indices for each type of model tested (see also Figures 2–4).

**Table 3:** Model fit indices for the exploratory and theoretical model.

	**Exploratory model**^**a**^	**Theoretical model**^**b**^	**Alternative model**^**c**^
χ^2^	123.92 (*p =* 0.021)	245.39 (*p* < 0.0001)	127.74 (*p* = 0.014)
χ^2^/*df*	1.318	2.504	1.345
CFI	0.987	0.936	0.986
AGFI	0.965	0.933	0.964
RMSEA	0.023 (0.009–0.03)	0.05 (0.042–0.057)	0.024 (0.011–0.034)
TLI	0.983	0.921	0.982
IFI	0.987	0.936	0.986
RFI	0.934	0.876	0.933
NFI	0.949	0.898	0.947
AIC	207.916	321.388	209.735
BIC	393.487	489.286	390.888
CAIC	435.487	527.286	431.888

*χ^2^, Chi Square; df, degrees of freedom; CFI, Comparative fit index; AGFI, Adjusted goodness of fit index; RMSEA, Root mean square error of approximation; TLI, Tucker-Lewis index; IFI, Incremental fit index; RFI, Relative fit index; NFI, Normed fit index; AIC, Akaike information criterion; BIC, Bayes information criterion; and CAIC, Consistent Akaike information criterion*.

a *The model was taken from the exploratory factor analysis*.

b *The model was taken from the first MMSI-2 validation (see Escolà-Gascón, 2020b)*.

c *The model was taken from the hypothesis that assumps the prediction of APP scales using the rest of factors*.

Both the empirical-exploratory model and the alternative model allow maintaining the null hypothesis of goodness of fit through the *Chi Square* statistic. In fact, for these two solutions, the AGFI (*adjusted goodness of fit index*) and the RMSEA (*root mean square error of approximation*), which take into account the degree of parsimony of the adjusted model, yielded favorable results, and the theoretical model offered a more parsimonious solution by including fewer parameters (see Figure 3). Unlike the comparative indices, the AIC and CAIC (*Akaike information criterion* and *Consistent Akaike information criterion*) and *Bayesian* (*Bayes information criterion* or BIC) indicators quantify the discrepancies observed between the variance-covariance matrix estimated from the parameters and the empirical variance-covariance matrix attributed to the data. According to these indices, the theoretical model showed the highest values, which means that it is the model that offers the most imbalance in relation to the other 2.

The exploratory-empirical model yielded the best fit indices, followed by the alternative model. However, the alternative model predicted anomalous phenomena (APP) with standardized regression weights below 0.3. Factors CPT, ASC and CPT predicted 18.3% of the variance of the anomalous phenomena evaluated by APP, which is a substantially low percentage compared to the original version (>50%) (see Escolà-Gascón, 2020a). Considering these results, in the Anglo-Saxon framework, it is appropriate to adjust the construct validity of the MMSI-2 according to the 5-factor solution and not the 4-factor solution.

### Reliability Analysis

In this study, the reliability of the scales and factors was examined using two types of methods: on the one hand, the *McDonald Omega* coefficients measured the internal consistency of the second-order factors, and on the other hand, Pearson's correlation coefficients were also used as reliability estimators between two equivalent but temporarily different applications. Table 4 shows the descriptive statistics for the factors of the extracted solutions and the *McDonald Omega* coefficients.

**Table 4:** Descriptive statistics and reliability coefficients for second order factors.

	**Factors**^**a**^	**Mean**	**Standard deviation**	**McDonald's Omega**
Empirical model	CPT (4)	126.97	32.99	0.705
	APP (4)	78.58	19.6	0.695
	IMA (4)	172.93	53.51	0.866
	ASC (2)	55.47	17.18	0.532
	OP (2)	31.32	9.72	0.608
Theoreticalmodel	CPT (6)	158.29	32.03	0.511

*CPT, Clinical Personality Tendencies; APP, Anomalous Perceived Phenomena; IMA, Incoherent Manipulations; ASC, Altered States of Consciousness; OP, Openness*.

a *The values in brackets are the number of variables per factor*.

**Figure 5:**
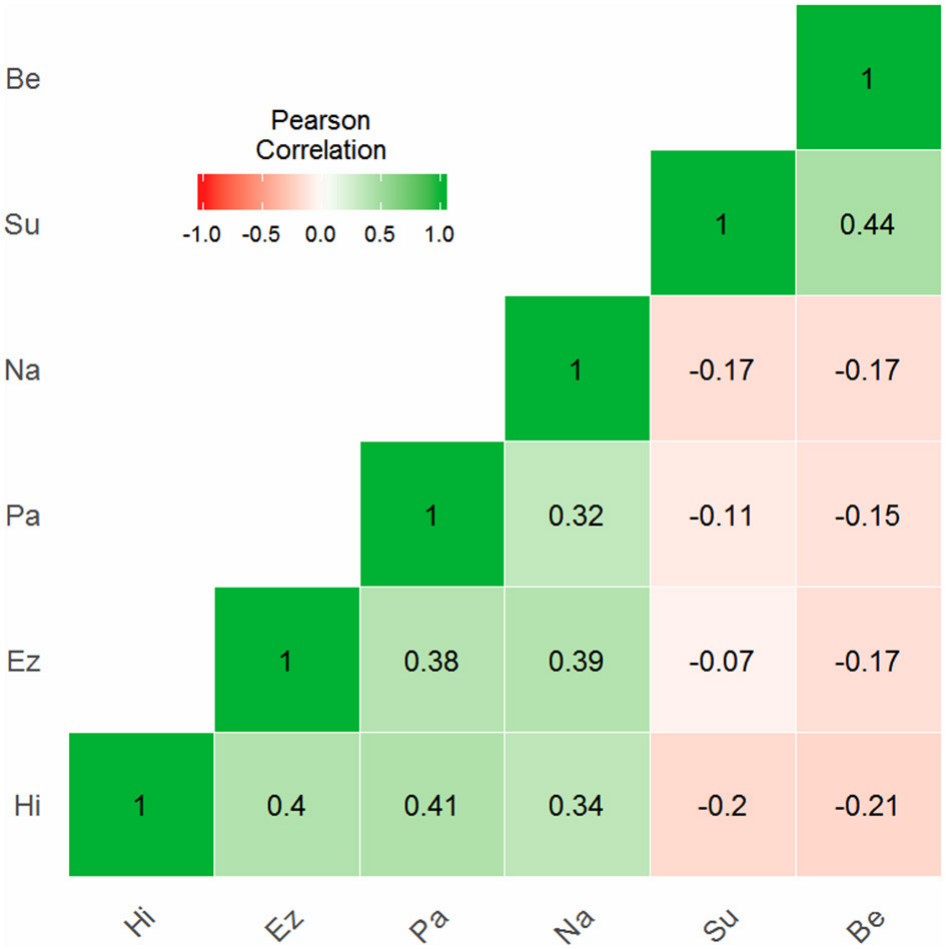
Heatmap and correlations between CPT scales (including Be and Su).

The omega coefficients were not especially high for most of the factors, with the exception of IMA and CPT, whose indices are above 0.7. When the CPT internal consistency was examined by including the Su and Be scales in this factor (see Figure 3), the factor obtained a poorer result (<0.55). The negative correlations between the Be-Su scales and the other indicators could explain this unexpected change. This is suspected because OP negatively correlates with CPT. However, this hypothesis can be tested by the correlation between Be-Su and the other scales that make up CPT in the theoretical model. Figure 5 shows a heatmap in which Be-Su is related to the CPT factor scales.

Tables 5, 6 contain descriptions for each scale and factor (given that this sample is limited to participants who responded promptly to the second application of MMSI-2). In this subsample, 142 subjects collaborated (30.9% were men and 23.3% were women). A total of 30.9% of the participants resided in Worthing, 10.3% in Portsmouth and 13% in Brighton. A total of 16.4% of the subjects completed secondary education, 20.2% received vocational training and 17.6% attended university studies.

**Table 5:** Test re-test coefficients and *t*-tests for paired groups.

**Comparisons pre and post**	**Mean**	**SD**	***r***	***t*-test** **(df = 141)**
(K) - Inconsistencies (pre-test)	28.6	9.54	0.613^*^	0.984*p* = 0.327
(L) - Inconsistencies (post-test)	27.92	9.26		
(L) - Lies (pre-test)	67.11	25.47	0.842^*^	−0.333*p* = 0.739
(L) - Lies (post-test)	67.5	24.61		
(F) - Fraud (pre-test)	58.46	20.38	0.815^*^	−0.545*p* = 0.87
(F) - Fraud (post-test)	59.02	19.58		
(Si) - Simulation (pre-test)	17.21	6.61	0.825^*^	−0.174*p* = 0.862
(Si) - Simulation (post-test)	17.27	6.44		
(Nt) - Neurasthenia (pre-test)	44.31	16.35	0.837^*^	−0.519*p* = 0.604
(Nt) - Neurasthenia (post-test)	44.7	15.11		
(Cs) - Substance Use (pre-test)	10.85	2.35	0.735^*^	1.476*p* = 0,142
(Cs) - Substance Use (post-test)	10.65	2.15		
(Su) - Suggestibility (pre-test)	19.7	6.55	0.841^*^	−1.032*p* = 0.304
(Su) - Suggestibility (post-test)	20.01	6.01		
(Be) - Thrill-Seeking (pre-test)	11.94	4.33	0.872^*^	−1.228*p* = 0.222
(Be) - Thrill-Seeking (post-test)	12.17	4.32		
(Hi) - Histrionism (pre-test)	32.47	11.72	0.786^*^	−0.208*p* = 0.836
(Hi) - Histrionism (post-test)	32.61	11.80		
(Ez) - Schizotypy (pre-test)	30.13	10.56	0.829^*^	0.095*p* = 0.925
(Ez) - Schizotypy (post-test)	30.08	10.64		
(Pa) - Paranoia (pre-test)	29.18	11.73	0.804^*^	0.499*p* = 0.619
(Pa) - Paranoia (post-test)	28.87	11.36		
(Na) - Narcissism (pre-test)	32.57	11.18	0.865^*^	0.311*p* = 0.756
(Na) - Narcissism (post-test)	32.42	10.46		

* *p < 0.0001*;

*SD, Standard deviation; r, Pearson correlation coefficient (also reliability coefficient); and df, degrees of freedom*.

**Table 6:** Test re-test coefficients and *t*-tests for paired groups (continuation Table 5).

**Comparisons pre and post**	**Mean**	**SD**	***r***	***t*-test(df = 141)**
(Pva) - Anomalous Visual/Auditory Phenomena (pre-test)	26.41	9.01	0.824^*^	−0.371*p* = 0.710
(Pva) - Anomalous Visual/Auditory Phenomena (post-test)	26.57	8.48		
(Pt) - Anomalous Tactile Phenomena (pre-test)	18.03	6.6	0.863^*^	−0.768*p* = 0.444
(Pt) - Anomalous Tactile Phenomena (post-test)	18.25	6.24		
(Po) - Anomalous Olfactory Phenomena (pre-test)	16.27	5.76	0.704^*^	−1.363*p* = 0.175
(Po) - Anomalous Olfactory Phenomena (post-test)	16.77	5.60		
(Pc) - Anomalous Cenesthetic Phenomena (pre-test)	17.06	5.31	0.747^*^	−1.314*p* = 0.191
(Pc) - Anomalous Cenesthetic Phenomena (post-test)	17.49	5.47		
(IMA) - Incoherent Manipulations (pre-test)	171.38	51.77	0.896^*^	−0.168*p* = 0.867
(IMA) - Incoherent Manipulations (post-test)	171.70	46.04		
(ASC) - Altered States of Consciousness (pre-test)	55.16	17.11	0.844^*^	−0.245*p* = 0.807
(ASC) - Altered States of Consciousness (post-test)	55.35	15.71		
(OP) - Openness (pre-test)	31.65	9.68	0.901^*^	−1.521*p* = 0.131
(OP) - Openness (post-test)	32.18	8.89		
(APP) - Anomalous Perceived Phenomena (pre-test)	77.77	18.63	0.875^*^	−1.721*p* = 0.087
(APP) - Anomalous Perceived Phenomena (post-test)	79.07	16.46		
(CPT) - Clinical Personality Tendencies (pre-test)	124.35	32.9	0.904^*^	0.310*p* = 0.757
(CPT) - Clinical Personality Tendencies (post-test)	123.98	30.13		

* *p < 0.0001*;

*SD, Standard deviation; r, Pearson correlation coefficient (also reliability coefficient); and df, degrees of freedom*.

These results did not show significant changes in the average scores for each scale and factor. All the variables included demonstrated significant, positive linear correlations, and in most cases, they were also high. The K scale showed the lowest correlation (*r*_*k*_ = 0.613). Only the APP factor possessed a critical level close to 0.01, but it was still not significant (*p* = 0.087). This indicated that the MMSI-2 examines behaviors whose longitudinal variability is reliable. Therefore, the high correlation indices and the non-significant critical levels ensured the stability of the scores and accept their reliability.

## Discussion

This paper outlines the English adaptation of the MMSI-2. This includes examination of internal validity and scale reliability. Regarding validity, analysis indicated that the MMSI-2 adequately represented subclinical psychological constructs and anomalous perceptions related to parapsychology. Concerning internal reliability, although McDonald omega coefficient was below the recommended lower limit (0.6) (see Hair et al., 2010), *test-retest trials* presented very favorable results, for both dimension and factor scores. Thus, the adapted MMSI-2 demonstrated satisfactorily validity and reliability.

### Conceptual Analysis Derived From the Theoretical Background

Before delving into the psychometric and statistical part of the MMSI-2, it is worth reflecting on the need and usefulness of an instrument such as this, applied in psychological evaluation and specifically in the field of anomalous phenomena. It is not incorrect to state that, at least for now, it is not possible to prove or verify the existence of “psi” phenomena (see French and Stone, 2014). Therefore, outside the experimental methodology, it is also not possible to contrast whether a given anomalous experience is truly a hallucination, a perceptual deformation, or a simple invention of the patient. In this context, the MMSI-2 represents a useful instrument for assessing abnormal experiences because it includes the main psychological indicators that predict these experiences. For example, a person who has had a “psi” experience and obtains high levels of schizotypy (Ez) may have experienced an attenuated hallucination of a psychotic nature rather than a delusion (Simmonds-Moore et al., 2019). However, if this person scored low on schizotypy and the other subclinical variables, it is possible that he or she would have had a non-pathological delusion.

As noted in the introduction, questionnaires that measure anomalous phenomena are scales that start from the apriorism belonging to model 1 (e.g., Wahbeh et al., 2019) or model 2 (e.g., Stefanis et al., 2002). In reality, it is not correct or objective that the investigations of model 1 affirm that there is scientific evidence in favor of parapsychological phenomena. Nor is it admissible that the investigations of model 2 comment on the following *naturalistic fallacy* (see Feldman, 2019): - parapsychological phenomena cannot exist because they are impossible at the scientific level (proposition A); - a subject tells me a parapsychological phenomenon (proposition B); therefore … - what the subject counts is a hallucination (fallacious conclusion). Only in proposition A can one already observe the *Aristotelian fallacy of affirming the consequent:* one cannot verify the “impossibility” of a phenomenon by contrasting hypotheses (see Popper, 2008). This is explained because the real basis of the MMSI-2 lies in this point: how to know if an anomalous experience is a hallucination, an illusion, an interpretation or a fraud. Following Escolà-Gascón's (2020b) criteria, an anomalous experience may be a hallucination when the participant obtains high scores (typical scores >50 or 60) on the K, Ez, Pa, and Cs scales. It will be an illusion or perceptual deception when the scores are elevated on the Hi, Na, Nt, Su, and ASC scales. It will be a subjective interpretation when the Si, Be, and Hi scales score very high. Likewise, it will be a fraud or a lie when the scores on the K, L, F, and Si scales are high.

The fact that a statistically valid model can be fitted in the structure of the MMSI-2 suggests that an alternative model is possible. This is discussed below.

### Methodological Analysis of the Results

The exploratory results of the initial EFA seem to coincide with the internal structure of the original statistical validation of the MMSI-2 (see Escolà-Gascón, 2020a,b; Escolà-Gascón and Gallifa, 2020). Unlike what was expected, the OP factor was novel because in the Spanish factorial solutions, only four macrofactors were retained. However, the grouping of scales offered by OP can be extrapolated to the classical theories of personality based on the “Big Five” model (see Goldberg, 1993). Both Be and Su are classified into multiple statistical and theoretical models of personality as two facets belonging to the *Openness* dimension (e.g., Costa and McCrae, 2008). The MMSI-2 is not a psychopathological test, and the items were written in such a way that they express attenuated subclinical contents in different degrees or levels that remain within the normative or non-clinical framework. This means that some scales of the MMSI-2 may have a certain correspondence with the factorial models of personality. For this reason, the Be and Su scales may compose the *Openness* or OP dimension. In future research, this could happen again with other MMSI-2 scales.

The scientific literature agrees that subjects believing in the existence of the paranormal tend to present elevated traits both in suggestion and in search of emotions (e.g., Jinks, 2019). However, in the results obtained, the OP factor correlates negatively with other MMSI factors. This seems unexpected, since in other studies, the factors IMA, CPT, ASC, and APP correlated positively with paranormal beliefs (see Irwin, 2009). In fact, the scales K, L, F, Si, Hi, Ez, Pa, Na, Cs, Nt, Su, and Be of the MMSI were obtained empirically (using EFAs and CFAs), and their reagents measure behaviors that, according to the scientific literature, see French and Stone (2014) for a review, are common in subjects who believe in the paranormal and claim to have had anomalous experiences (see also Escolà-Gascón, 2020b). If this is so, it seems strange that the correlations between OP and the other factors are negative, especially the covariation with APP. In the Spanish version, the correlations are positive between all factors. This raises the debate about whether the predictor variables of paranormal beliefs and experiences could have different effects according to the sociocultural environment from which the participants come. Is it likely that the culture and the educational model promote different interpretations of the items in these two scales? As suggested by Brown (2015), to assess this the factorial invariance of the model of 5 must be analyzed comparing two equivalent samples from different cultural environments or countries.

An important detail is that in the three models examined, the factors were related to each other, which seems to indicate that the anomalous phenomena (APP) do not have an independent operation with respect to the other variables. However, although the models in Figures 2, 4 provide correlations close to zero (one is between ASC and IMA and the other is observed between ASC and CPT), the model in Figure 3 only maintains this trend for the relationship between ASC and IMA. The standardized covariance between ASC and CPT is equal to 0.104. The fact that the 4-factor model shows a higher correlation between CPT (*Clinical personality tendencies*) and ASC (*Altered States of Consciousness*) can be explained by the inclusion of the Su and Be scales in the CPT factor. As shown in Figure 5, these two scales correlate negatively with the other dimensions of the same factor. Therefore, it is likely this compromises the internal consistency of CPT (see Table 4).

Nevertheless, at the same time, it could generate an increase in the covariance between CPT and ASC. This would have an impact on CPT interpretation: on the one hand, in Figures 2, 4 CPT could indicate attenuated clinical tendencies of the personality, by including behaviors that are not necessarily psychopathological but their qualitative content if included in the systems clinical classification (see *Diagnostic and Statistical Manual of Mental Disorders*, DSM-5). On the other hand, in Figure 3, the interpretation of CPT is more complex, since it could describe non-pathological contents, but Be and Su would have a pre-disposition to the clinical because they are included in the same group as Hi, Ez, Pa, and Na. Whereas, some conventional personality questionnaires - for example the NEO-PI-R, from Costa and McCrae (2008) - do not have an applicability in the clinical evaluation, other questionnaires also of the personality - for example the 16PF of Cattell (1946) - they do have value in psychopathological terms (see Karson et al., 2003). This allows us to consider the possibility that MMSI-2 may also have clinical utility, especially for the CPT and OP factor scales. It would be advisable to test the 4- and 5-factor model in non-clinical samples (without a psychiatric history) and clinical samples (with a formally diagnosed history), with the objective of analyzing the factorial invariance of each of the factors and their scales. Is it possible that OP represents a different construct when it is applied in a clinical sample?

It should also be questioned why the ASC, CPT, and IMA factors predict only 18.3% of the variance of anomalous phenomena (APP). In the original version and using the same factors, this explained variance increases substantially to 51.2% (see Escolà-Gascón, 2020a). Again, this suggests that the interpretation of the MMSI-2 scales may have different connotations when the cultural environment changes. This does not have to directly affect the construct validity of the MMSI-2. To contrast the possibility of biased and different interpretations (in addition to examining factorial invariance), the analysis of the *Differential Item Functioning* (hereafter DIF) could also be considered (see DIF, Abad et al., 2015). In reality, within the context of parapsychological beliefs and experiences, it would not be the first time that a test presents biases or DIF when comparing the responses between believing and non-believing subjects in the existence of the paranormal (see Lange et al., 2000). However, if this proposal were applied, the specific items of the scales to be evaluated should be selected, since analyzing the presence or absence of DIF for the 174 items of the MMSI-2 is somewhat costly at the logistical level. As an alternative, logistic regression could be used using the direct scores of the classes as predictive variables.

Regarding the reliability indices, it should be noted that the internal consistency of the ASC and OP macrofactors is not high because omega coefficients were close to the 0.6 cut-off point. IMA offers the highest value, and the other factors yield acceptable or questionable results. This questions the accuracy of the factor scores in the individual interpretation of the profiles. Thus, although scales have already been defined for the Spanish population, the English standardization of the scores would not be recommended until higher internal consistency indices in the ASC and OP macrofactors were obtained. For the other scale dimensions and factors, a first proposal for the standardization of direct scores could be initiated.

Although the low reliability indices based on internal consistency already represent a limitation for the use of the MMSI in the professional practice of psychological evaluation, in statistical terms, the reliability of the questionnaire can be accepted if the *test-retest* trials applied are taken into account. Both internal and longitudinal consistency represent two empirical markers of the same psychometric property: reliability. It would be ideal to accept both types of reliability (internal and longitudinal), but the acceptance of one already confirms the reliability of the test in statistical terms (although the good results of one do not replace the shortcomings of the other) (see Abad et al., 2015).

The K scale showed the lowest correlation (although it was also higher than 0.6). This fact may be due to the type of content and items included in this dimension. This is a scale that examines the presence of logical inconsistencies in the responses of the participant. This allows (1) to know if the evaluated subject answers randomly to the test questions, (2) if he correctly understands the statements and (3), his collaborative predisposition toward the evaluation. These three characteristics could yield a temporal variability in K more independent with respect to the other scales. It is possible that this has affected the covariability of the scores and, therefore, their temporal consistency. However, the correlation obtained in this scale is acceptable for this type of test (see Abad et al., 2015).

### Criticisms and Limitations

At least in the sample used, it can be stated that the macrofactors were positively related to APP. That is, as a subject perceives anomalous phenomena, it is also possible to present correlative traits in the other factors of the MMSI. According to what traits the subject presents, the perceived anomalous phenomena could be validated as hallucinatory phenomena, perceptual deformations, cognitive-social biases, belief systems, or as unexplained behaviors. In this context, the psychological attributes related to psychosis (e.g., the Ez, Pa, K, and Cs scale of the MMSI-2) (see Fonseca-Pedrero et al., 2011), combined with high scores in APP, support the hallucinatory value of the perceived parapsychological phenomena (and therefore, they should no longer be called “anomalous”). The same hypothetical logic could be applied to perceptual deformations and other typologies that define the “supposed” anomalous phenomena.

However, with such a low percentage of variance, it seems advisable to offer decision criteria that specify what combinations of scales it would be possible to discriminate between a hallucination, a bias, a perceptual deformation, etc. It should not be forgotten that the main objective of this research was the psychometric examination of the validity and reliability of the English adapted MMSI-2. Therefore, the analysis of the quality and predictive quality of the ASC, CPT and IMA factors on APP should be tested using cut-off points and analysis of *receiver operating characteristic* (ROC) curves.

Taking into account that the believing subjects in the “supernatural” tend to present higher levels in the different scales that measure hallucinations and perceptual deformations with respect to non-believers (see Matute et al., 2011; Griffiths et al., 2018; Torres et al., 2020; Wright et al., 2020), a possible way for the covariation between the macrofactors to increase would be by replicating the CFA for the 5-factor model only with subjects believing in the existence of the paranormal. It seems likely that the participants of this sample do not believe in the existence of the paranormal with the same intensity as the subjects of the Spanish samples. This could generate a bias in the APP scores that would harm the correlations with the other factors. Thus, “beliefs in the existence of the paranormal” could be a strange variable that should be controlled in future research.

Nevertheless, the construct validity of the model belonging to Figure 2 offers sufficient reasons to continue reviewing the psychometric properties of MMSI-2 and the application of the hypothetical empirical-statistical model as an explanation of anomalous phenomena in parapsychology, but from a rational and psychological perspective.

A relevant limitation is also related to the theoretical interpretation of the OP factor. It has already been mentioned in the previous subsection the possibility - although it is not a proven fact - that in English culture scales and macrofactors could have different meanings in relation to social interpretations from the Spanish-speaking culture. This would involve reviewing the factorial invariance and the possibility of DIF in some of the items or scales of the MMSI-2. It should be taken into account that the OP factor represents a first hypothetical classification extracted from the statistical evidence from the applied EFA, but it should not be accepted as a conclusive macrofactor, since in other EFAs and CFAs, this respective macro was not retained. -factor.

The fact of not saving the responses of the subjects to each item of the MMSI and using the direct calculation of the scores required estimating the internal consistency only for the second-order factors or macrofactors. This way of proceeding is not ideal, but it is not incorrect at the psychometric level (e.g., Arribas, 2011). In reality, the probability that the measurement model of a test obtains a good fit increases when the structural model applied to the scales also shows a correct fit (see Brown, 2015). This is due to mathematical reasons (see Mulaik, 2018) and because the structural model validates not only the quality of the measurements - whose test scales would be incorporated into the structural equations as observable variables -, it also validates the underlying theoretical construct. It is a top-down methodological process: valid constructs that form valid theories must offer guarantees that prove the validity of the respective measurements (see Gorsuch, 1983).

Therefore, in the MMSI-2, the individual responses were originally recorded in the online software but were not saved because it was not intended to examine the ordinal responses of the items but the factorial and structural model of the test itself. In addition, if the answers are ordinal, they should not technically be analyzed using factorial procedures, which require that the variables be quantitative (e.g., Mulaik, 2018). Working with the scales directly saves the dilemma of how to save this class of situations, in which it is debated how to quantitatively treat variables whose values are discrete-ordinal. A possible solution would have been the use of the *polychoric* correlations applied in the responses of the items, but as has already been justified, this was not part of the objective of this research.

Finally, we would like to highlight a limitation related to the application of the post-tests. We allowed participants a flexible margin of 10 days to answer the post-tests. This was done because not all participants could answer the post-tests in a timely manner. We did not want to put additional pressure on the participants and for this reason we offered them a few flexible days. However, this difference in the dates of the post-tests could have generated a variability that would have minimally biased the test-retest reliability coefficients. If this strategy is used in future research, we recommend analyzing the effects of this date-related variability. However, being only 10 days, we also believe that the variability will have had minimal effects on the results. As an adjunct, we also suggest that future applications take into account the ways of application of this assessment test. It is likely that there is also variability regarding to the format of application of the MMSI-2 (i.e., pencil-paper or digital format). In this research the applications were exclusively digital. Thus, it would also be useful to analyze whether there are differences between responses collected both conventionally (pencil-paper) and those obtained online.

## Conclusions

The *Multivariable Multiaxial Suggestibility Inventory-2* (MMSI-2) presents a valid internal structure formed by five factors: *Clinical Personality Tendencies* (CPT), *Anomalous Perceived Phenomena* (APP), *Incoherent Manipulations* (IMA), *Altered States of Consciousness* (ASC) and *Openness* (OP). This model belonging to the MMSI-2 is called *empirical-statistical* for two reasons: (1) because both the scales and the factors were extracted using statistical-factorial techniques and (2) because the scores of the scales and factors represent empirical markers of the behavior that allow us to correlate and predict anomalous phenomena, including “psi” phenomena (APP). The CPT, IMA, and ASC factors are correlated and explain 18.3% of the APP macrofactor. However, more than 50% of the variance of anomalous phenomena remains to be explained. This result contrasts with the Spanish version of the MMSI-2, in which these same factors predict anomalous phenomena with a weight of 51.2% of the variance. It is concluded that the low explained variance obtained in this research is because the subjects of the sample were not believers in the existence of the paranormal. This could affect the covariation between the factors, causing some of them to have a more independent statistical behavior.

The MMSI-2 offers reliable and stable scores over time, whose longitudinal consistency is guaranteed for at least 160 days after the first application. The reliability relative to the internal consistency of the scores belonging to the macrofactors was not very high and for this reason should be reviewed in future research.

The empirical-statistical model should be analyzed again to review the predictive value of the factors CPT, ASC, and IMA on APP. However, this research offers results that prove the validity and reliability of the MMSI-2 in the English population and support the relationship between APP and the other factors.

## Data Availability Statement

The raw data supporting the conclusions of this article will be made available by the authors, without undue reservation.

## Ethics Statement

The studies involving human participants were reviewed and approved by The Committee of Ethical Guarantees of Ramon Llull University. The patients/participants provided their written informed consent to participate in this study.

## Author Contributions

ÁE-G conceived and planned the study, collected the sample, performed the statistical analyses, and wrote the manuscript in consultation with ND. JG supervised the project. All authors contributed to the article and approved the submitted version.

## Conflict of Interest

The authors declare that the research was conducted in the absence of any commercial or financial relationships that could be construed as a potential conflict of interest.
